# A case of retroperitoneal fibrosis secondary to chronic periaortitis

**DOI:** 10.1259/bjrcr.20190011

**Published:** 2019-04-29

**Authors:** Mariam A. Omar, Naushad Hassan Karim, Saeed samnakay

**Affiliations:** Aga khan university hospital, Nairob, Kenya

## Abstract

Retroperitoneal fibrosis is a rare inflammatory disorder causing increased fibrotic deposition in the retroperitoneum, often leading to ureteral obstruction. We present a case report of a 33-year-old male with 2-month history of back pain. Initial imaging showed thickening around the infrarenal aorta. Six months later the patient presented with renal failure; a CT abdomen revealed extensive soft tissue mass around the aorta resulting in ureteral obstruction. Histology results of biopsy of the soft tissue mass revealed retroperitoneal fibrosis. It is important for clinicians to treat periaortitis early as this can prevent progression to retroperitoneal fibrosis which can cause severe secondary complications such as renal failure from ureteral obstruction.

## INTRODUCTION

Retroperitoneal fibrosis is a rare inflammatory disorder first described in 1948^1^ and consists of increased fibrotic deposition surrounding the major vessels and organs located within the retroperitoneum.

We present a case of idiopathic RPF in a young male which presented initially as periaortitis with low back pain and weight loss. Six months later the patient presented with renal insufficiency secondary to ureteric obstruction.

## Case report

A 33-year-old African male with history of smoking, presented with low back pain and weight loss of 9 kg within the preceding 11 months. The pain was continuous, blunt in character, worse with walking and radiated to the loins and thighs bilaterally. Past medical history was unremarkable.

On physical examination, the patient was normotensive. Examination of the cardiovascular and respiratory systems was unremarkable. Arterial pulsations of the lower extremities were palpable bilaterally.

**Figure 1.**
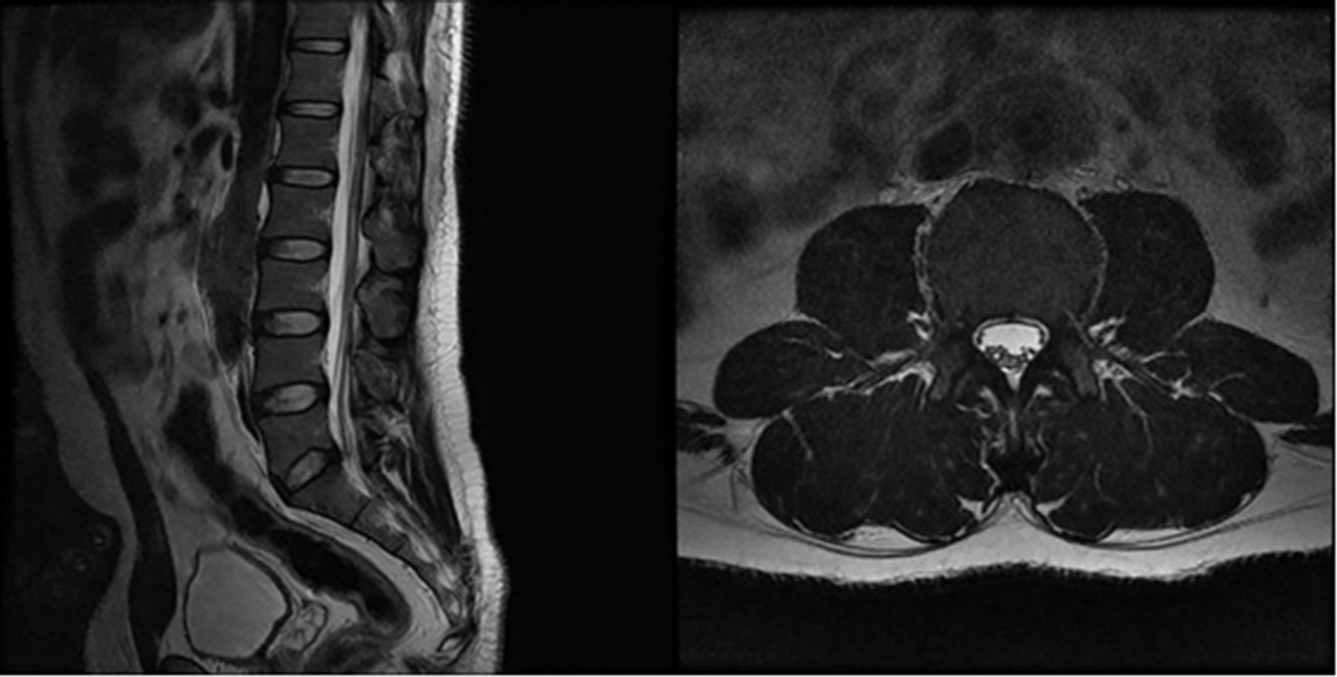
Sagittal and axial T2W images of the lumbar spine demonstrating circumferential soft tissue thickening around the infra renal aorta.

In view of the severe back pain an MRI (GE SIGNA EXCITE 1.5T) lumbar spine was done which revealed both T1W and T2W isointense periaortic thickening (Figure 1). No disk pathology was identified.

**Figure 2.**
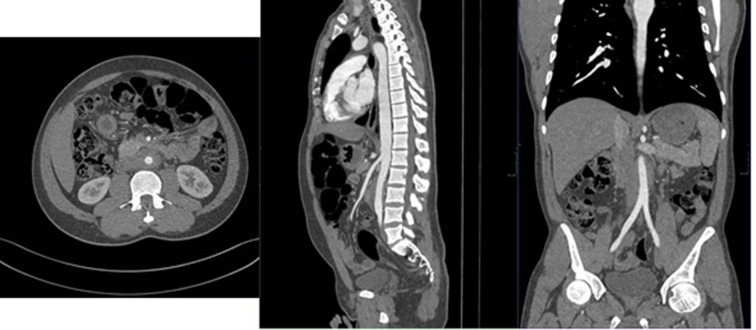
Axial, sagittal and coronal CT aortogram images demonstrating circumferential soft tissue thickening around the infra renal aorta.

CT Aortic angiogram (SIEMENS SOMATOM DEFINITION FLASH 256 SLICE DUAL ENERGY) was done which revealed thickening of the anterior and lateral aspect of the aorta No aneurysmal dilation or distal narrowing were seen (Figure 2).

A diagnosis of periaortitis was entertained at that time and further tests were recommended to rule out an autoimmune process. However due to financial constraints the patient decided to seek herbal treatment only to present with severe back pain and lower limb swelling 6 months later.

**Figure 3.**
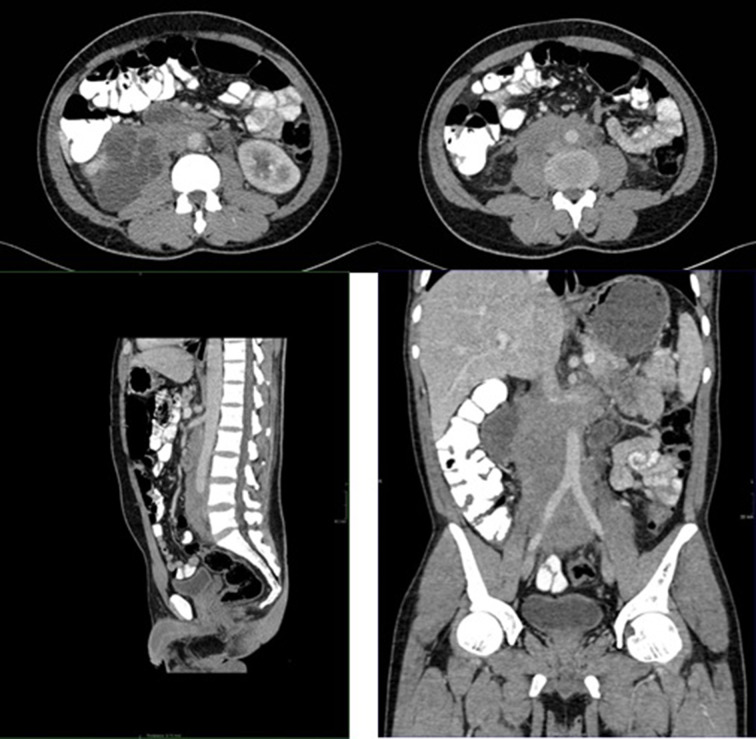
Axial, sagittal and coronal images of the abdomen demonstrating irregular retroperitoneal soft tissue mass centred around the aorta with medial displacement of the bilateral ureters and bilateral hydroureters. Interval increase in extent is also demonstrated.

A repeat CT of the abdomen and pelvis revealed extensive soft tissue thickening concentrically around the abdominal aorta, both iliac arteries, the inferior vena cava, and the ureters bilaterally (Figure 3). There was bilateral hydronephrosis and proximal hydroureter. The ureters were noted to be displaced medially bilaterally. Differential diagnoses of retroperitoneal fibrosis, lymphoma and retroperitoneal tumour/sarcoma were entertained at this time.

**Figure 4.**
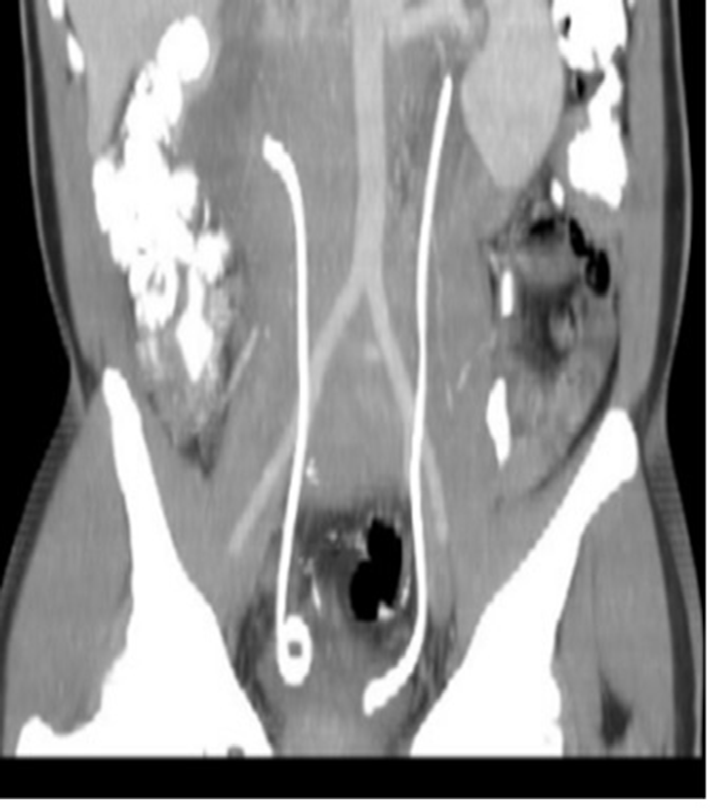
Coronal reconstruction of CT abdomen and pelvis demonstrating JJ stents *in situ.*

The patient had elevated creatinine (192 µmol/l) from the ureteral obstruction and bilateral Double-J stents were placed to relieve the obstruction. (Figure 4). Subsequent lab tests showed a decrease in serum creatinine (111 µmol/l).

**Figure 5.**
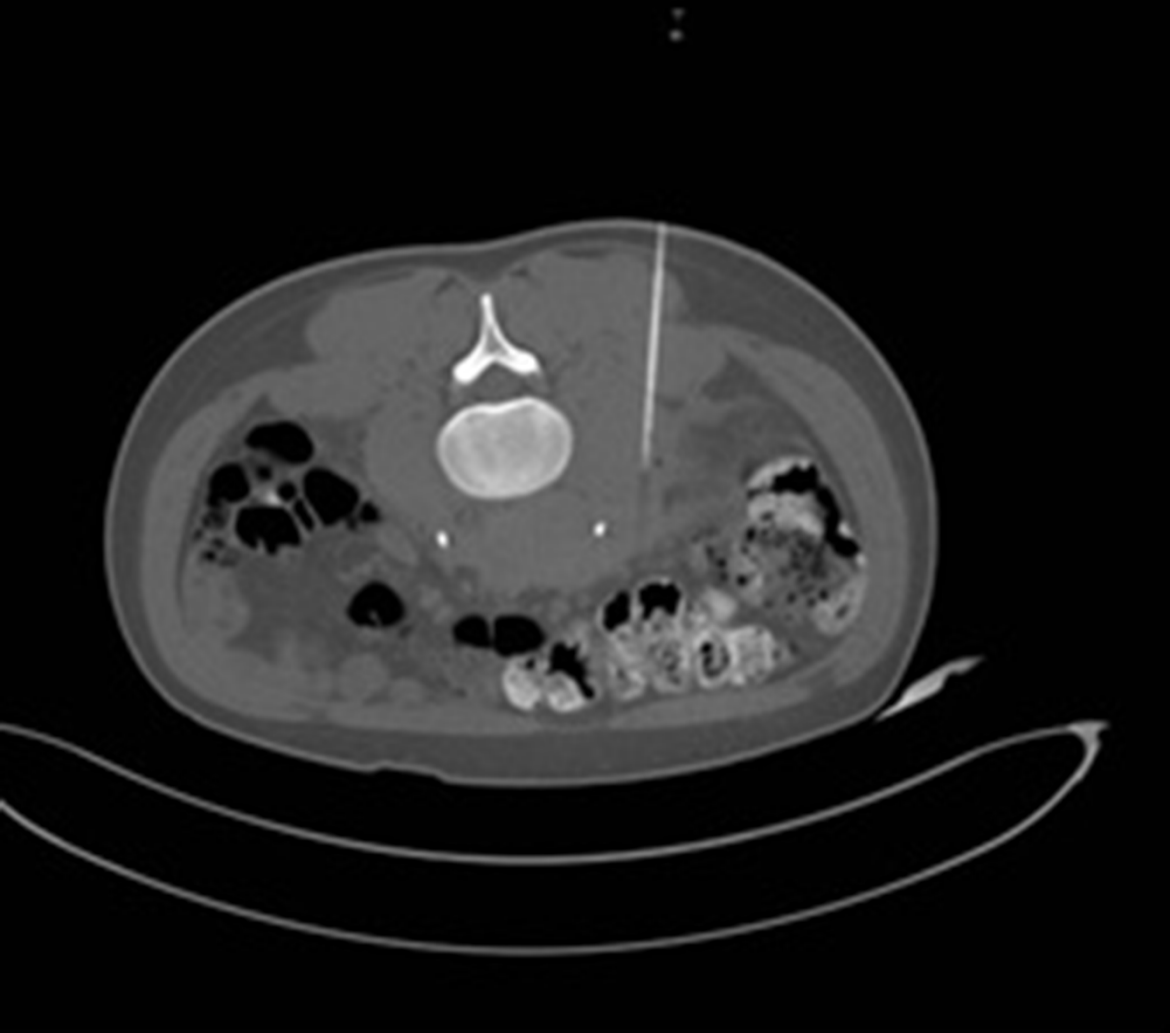
Axial image demonstrating CT guided core needle biopsy of the retroperitoneal mass.

A CT guided biopsy of the soft tissue mass (Figure 5) was performed for definitive diagnosis.

### Histological diagnosis

The biopsy showed scant fragments composed of fibrous tissue and collagen interspersed plasma cells and eosinophils. Immunohistochemistry showed cells to be positive for Vementin. Punkeratin, S100 and CD 34 were negative. No necrosis or mitoses was identified. A diagnosis of retroperitoneal fibrosis was arrived at.

**Figure 6.**
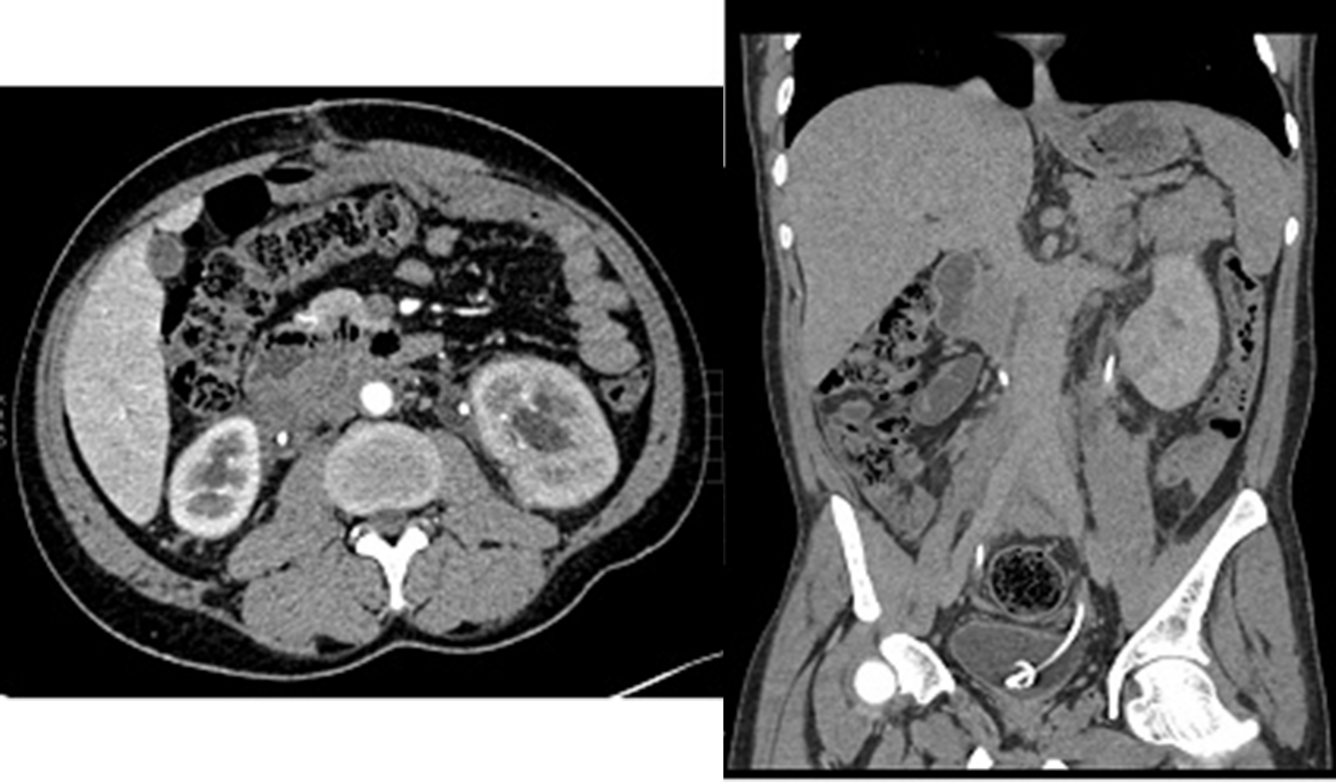
CT abdomen 6 months post treatment showing marked reduction in periaortic soft tissue. Stents *in situ* with reduction in extent of hydronephrosis.

The patient was discharged home on steroids and follow up imaging after 6 months was recommended(Figure 6).

## Discussion

Retroperitoneal fibrosis entails the presence of a fibro-inflammatory tissue which surrounds the abdominal aorta and its branches and extends into the retroperitoneum to envelop neighbouring structures such as the ureters.^2^

It can be broadly classified as either idiopathic or secondary to a cause. Methylsergide, an ergot prescribed for migraine headache causes 12% of cases.^3^ Small foci of metastatic malignancy that elicit a fibrotic reaction account for another 8–10% of cases. About 15% percent of patients have additional fibrosing processes, including mediastinal fibrosis, Riedel fibrosing thyroiditis, sclerosing cholangitis and fibrotic orbital pseudotumours. Two thirds of the cases are idiopathic, however, the term idiopathic may be wrong since it is now believed to be secondary to advanced atherosclerosis and is likely an autoimmune process. However, our patient did not have atherosclerotic plaques in the CT aortic angiogram. RPF is twice as common in males when compared to females and is commonly seen between the ages of 30 and 60 years.^1^

Because the symptoms are non-specific and there are no specific laboratory tests, retroperitoneal fibrosis is commonly diagnosed only after renal failure has been established. However, if diagnosed and managed early, benign forms of retroperitoneal fibrosis have a good prognosis.^4^ Often the diagnosis relies heavily on radiologic findings.^5^Biopsy is also often necessary to confirm the diagnosis, especially if there is lack of experience with diagnosis and management of retroperitoneal fibrosis, imaging findings or clinical/laboratory suspicion of an underlying malignancy, atypical location (pelvic, perirenal, peripancreatic) or absence of response to immunosuppressive therapy.^4^ However, histological characterization of idiopathic retroperitoneal fibrosis is not well defined and protocol not standardized in terms of immunohistochemistry panel needed.^6^

Imaging plays a key role in the diagnosis and follow up in retroperitoneal fibrosis and should be performed in patients with unexplained, persistent back, flank or abdominal pain.^6^ultrasound finding are non-specific and in severe cases hydronephrosis is noted. Contrast enhanced CT abdomen and pelvis is able to diagnose retroperitoneal fibrosis however it cannot differentiate between benign and malignant etiology of RPF.MRI can also be used to assess the extent of disease and its relationship with adjacent organs without the need of contrast agents hence it can be used in patients with renal failure.^6^It cannot however differentiate between benign and malignant RPF.FDG PET imaging shows increase uptake in benign and malignant RPF hence its role is limited to follow-up.^6^

As our case highlights, it is important to recognise periaortitis early and institute early treatment to prevent progression to retroperitoneal fibrosis with its attendant complications such as renal failure. It is also important to be able to differentiate between retroperitoneal fibrosis from lymphoma or retroperitoneal sarcoma. The most important imaging clues for this are periaortic thickening in the early phase of the disease, irregular soft tissue around the aorta without its anterior displacement and medialisation of the ureters with external compression resulting in hydroureteronephrosis,^7^ in contrast to lymphoma which may have a more nodular appearance and may cause anterior displacement of the aorta.^8^ However, anterior displacement of the aorta in retroperitoneal fibrosis is suggestive of malignancy and biopsy is advised when the aetiology is in doubt.

There are no guidelines on the treatment of idiopathic RPF however therapy is dependent upon the stage of the disease at diagnosis. Insertion of DJ endoureteric catheters are indicated if ureteric obstruction is present.

Treatment with immunosuppressive drugs in the management of idiopathic RPF has also shown disease regression. corticosteroids have also been used in the management of idiopathic RPF with improved general symptoms and radiographic findings.^9^The outlook is usually good with early diagnosis and management ;delay in diagnosis can cause severe complications, such as end-stage renal failure. Our patient is being managed with corticosterioids.

In our case, the idiopathic RPF presented as low back pain and weight loss with periaortitis identified on initial imaging. It is therefore paramount for clinicians to consider RPF as a potential complication of aortitis even in young patients as early initiation of treatment may halt its progression.

## Learning points

- Identifying periaortitis on imaging and institute early treatment to prevent progression to retroperitoneal fibrosis with its attendant complications such as renal failure.
- How to differentiate lymphoma from retroperitoneal fibrosis
- Biopsy is warranted in cases where the aetiology is not clear
